# On-line range verification for proton beam therapy using spherical ionoacoustic waves with resonant frequency

**DOI:** 10.1038/s41598-020-77422-2

**Published:** 2020-11-23

**Authors:** Taisuke Takayanagi, Tomoki Uesaka, Yuta Nakamura, Mehmet Burcin Unlu, Yasutoshi Kuriyama, Tomonori Uesugi, Yoshihiro Ishi, Nobuki Kudo, Masanori Kobayashi, Kikuo Umegaki, Satoshi Tomioka, Taeko Matsuura

**Affiliations:** 1 Graduate School of Biomedical Science and Engineering, Hokkaido University, North-13 West-8, Kita-ku, Sapporo, Hokkaido 060-8628 Japan; 2 Hitachi Ltd, 1-1 7-chome, Omika-cho, Hitachi-shi, Ibaraki, 319-1292 Japan; 3 Graduate School of Engineering, Hokkaido University, North-13 West-8, Kita-ku, Sapporo, Hokkaido 060-8628 Japan; 4 Department of Physics, Bogazici University, Bebek, Istanbul, 34342 Turkey; 5 Institute for Integrated Radiation and Nuclear Science, Kyoto University, Kumatori, Osaka 590-0494 Japan; 6 Faculty of Information Science and Technology, Hokkaido University, North-14, West-9, Kita-ku, Sapporo, Hokkaido 060-0814 Japan; 7 Planetary Exploration Research Center, Chiba Institute of Technology, Narashino, Chiba 275-0016 Japan; 8 Faculty of Engineering, Hokkaido University, North-13 West-8, Kita-ku, Sapporo, Hokkaido 060-8628 Japan; 9 Proton Beam Therapy Center, Hokkaido University Hospital, North-15 West-7, Kita-ku, Sapporo, Hokkaido 060-8638 Japan

**Keywords:** Radiotherapy, Biomedical engineering

## Abstract

In contrast to conventional X-ray therapy, proton beam therapy (PBT) can confine radiation doses to tumours because of the presence of the Bragg peak. However, the precision of the treatment is currently limited by the uncertainty in the beam range. Recently, a unique range verification methodology has been proposed based on simulation studies that exploit spherical ionoacoustic waves with resonant frequency (SPIREs). SPIREs are emitted from spherical gold markers in tumours initially introduced for accurate patient positioning when the proton beam is injected. These waves have a remarkable property: their amplitude is linearly correlated with the residual beam range at the marker position. Here, we present proof-of-principle experiments using short-pulsed proton beams at the clinical dose to demonstrate the feasibility of using SPIREs for beam-range verification with submillimetre accuracy. These results should substantially contribute to reducing the range uncertainty in future PBT applications.

## Introduction

Radiotherapy permits the treatment of patients as outpatients and can help them maintain a high quality of life; thus, it is widely chosen for cancer treatment. In particular, the treatment outcome expectations based on proton beam therapy (PBT) have increased in recent years, and a number of patients have their cancers treated successfully using PBT^1^. PBT is based on the physical properties of proton, which cause the formation of a sharp Bragg peak at the end of the beam range, implying that no dose is received downstream of the peak. In general in radiotherapy, completely protecting the healthy tissue surrounding the tumour against radiation exposure is difficult, which leads to the risk of side effects, such as ulcers and radiation pneumonitis. However, unlike conventional X-ray therapy, PBT can confine the radiation dose to the tumour and spare the surrounding healthy tissue due to the Bragg peak, which is advantageous^2,3^. The precision of PBT largely relies on the accuracy of the prediction of the Bragg peak position. However, in current clinical practice, within the human body, the range of the uncertainty has been estimated to be 1–3% (e.g. about 1 cm at a nominal range of 30 cm), and significant efforts have been made within the past decade to reduce this uncertainty^4,5^.

Range measurement during beam delivery (‘on-line range verification’) is a very active area of research in this context. There are three main approaches for the measurement: positron emission tomography (PET)^6^, prompt gamma-ray (PG) detection^7^ and ionoacoustic detection. PET and PG detection estimate the beam range by measuring the vertices of gamma-ray emission due to the nuclear inelastic reactions between protons and nuclei in media. Despite intensive research efforts, these techniques still require bulky gamma-ray detectors to be placed around the patient, and direct range observations are not possible since the nuclear inelastic cross-section falls to nearly zero at the proton range^6^.

In contrast, the ionoacoustic wave detection is a direct approach for measuring shock waves generated from the entire volume of dose deposition, especially the Bragg peak. The detection system comprises a hydrophone, which is more affordable than an expensive gamma-ray detector. This method has historically been explored for neutrino detection in deep-sea^8^ or high-energy particle physics^9^. In PBT applications, several simulation-based studies have been performed, focusing on analysis in a simple water medium^10–13^ and in prostate and liver cancer patients^14^. Moreover, experimental studies have been performed, and positive results have been demonstrated using a linac^15^, a tandem accelerator^16^, a synchrotron^17–20^, laser-plasma accelerator^21^, and clinical-use accelerators, such as an isochronous cyclotron^22^ and a synchrocyclotron^23^. However, compared to PET and PG detection, the use of the ionoacoustic wave detection has not been much explored because of the weakness of the pressure wave (of the order of millipascals) available during clinical beam delivery^22^. Signal averaging of repeated measurements is required, which is not practicable in clinical settings.

**Figure 1:**
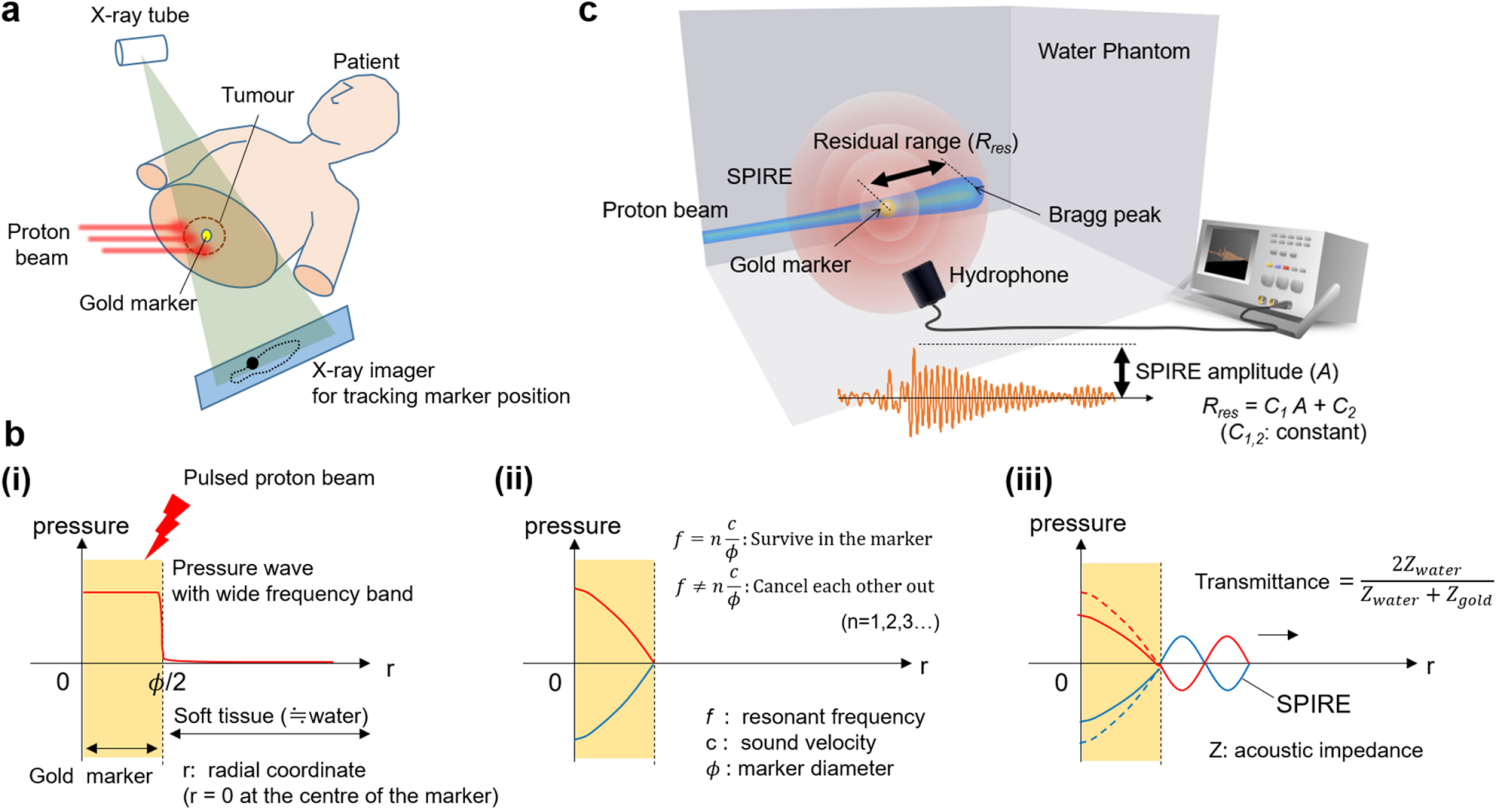
Proton beam range detection using SPIRE measurement. (**a**) Schematic representation of patient positioning during proton beam delivery using a gold fiducial marker. Spherical gold markers of 2 mm diameter are inserted in or near to the tumour and constantly monitored by X-ray fluoroscopy during beam delivery. If two sets of fluoroscopies are used, the marker’s three-dimensional coordinates can be obtained. (**b**) Schematic drawing of the SPIRE emission. (i) Upon the injection of the proton beam, the material properties of the gold act as a strong pressure source compared to the surrounding biological tissue. (ii,iii) However, due to the large difference in acoustic impedance between the gold and the tissue, most of the pressure waves will reflect back at the boundary and decay quickly as they superpose within the marker. Only waves with a frequency equal to the resonant frequency of the marker survives in the marker and spill outward into the surrounding tissue. This resonant frequency is 1.62 MHz for a marker with a diameter of 2 mm. (**c**) Schematic drawing of the SPIRE detection and range estimation. The SPIREs are observed by a hydrophone less than 100 μs after the beam delivery. Since the SPIRE amplitude is correlated with the residual beam range, *R*_*res*_, at the marker and the marker position is constantly monitored by the fluoroscopy system in clinical cases, the beam range can be estimated if the relation between the SPIRE amplitude and *R*_*res*_ is known beforehand.

Recently, an alternative acoustic range-detection method has been proposed^24^; it uniquely exploits the biocompatible gold fiducial markers that are inserted in tumours for accurate positioning of patients during treatment beam delivery (Fig. 1a)^25–29^. As illustrated in Fig. 1b, when the spherically shaped gold marker is hit by the pulsed proton beam, the marker will emit a spherical acoustic wave that has a frequency solely determined by its diameter: $f=cn/\phi \left(n=\mathrm{1,2},\dots \right)$, where $c$ and $\phi$ are the sound velocity in gold and the diameter of the gold marker, respectively. For instance, for  $\phi$ = 2 mm, which is the size currently used in clinics, $f$ will be 1.62 MHz. This wave is referred to as a spherical ionoacoustic wave with resonant frequency (SPIRE). SPIRE amplitude is linearly correlated with the residual beam range at the marker (*R*_*res*_) as long as the marker is positioned close to the range, which allows the estimation of the Bragg peak position using acoustic measurements (Fig. 1c). As presented in a convincing numerical simulation^23^, if a short-pulsed proton beam (pulse width ~ 100 ns) is incident on the marker, the magnitude of the resonance wave should reach several Pascal for a beam of ~ 10^8^ protons, which is the number often delivered in a single spot beam using the spot-scanning beam delivery technique^30^. Although such a relatively large pressure wave can also stem from a Bragg peak with the same order of short pulse width^18^, the SPIRE method is more advantageous than the conventional acoustic range detection method as the frequency of the SPIRE does not depend on the incident proton energy, and thus, the sensitivity of the detection system can be improved by focusing on a single frequency.

In this study, we demonstrate this method in a proof-of-principle experiment using a short-pulsed proton beam produced by a fixed-field alternating gradient accelerator (FFA), which is one of the candidate accelerators for future PBT^31^. Using a specially customised hydrophone and amplifier, the resonance wave is detected with a sufficiently large signal-to-noise ratio, and the Bragg peak position is identified from a single measurement. The success of the acoustic range detection with submillimetre accuracy should substantially contribute towards reducing the range uncertainty in future PBT applications.

## Results

### Experimental setup

**Figure 2:**
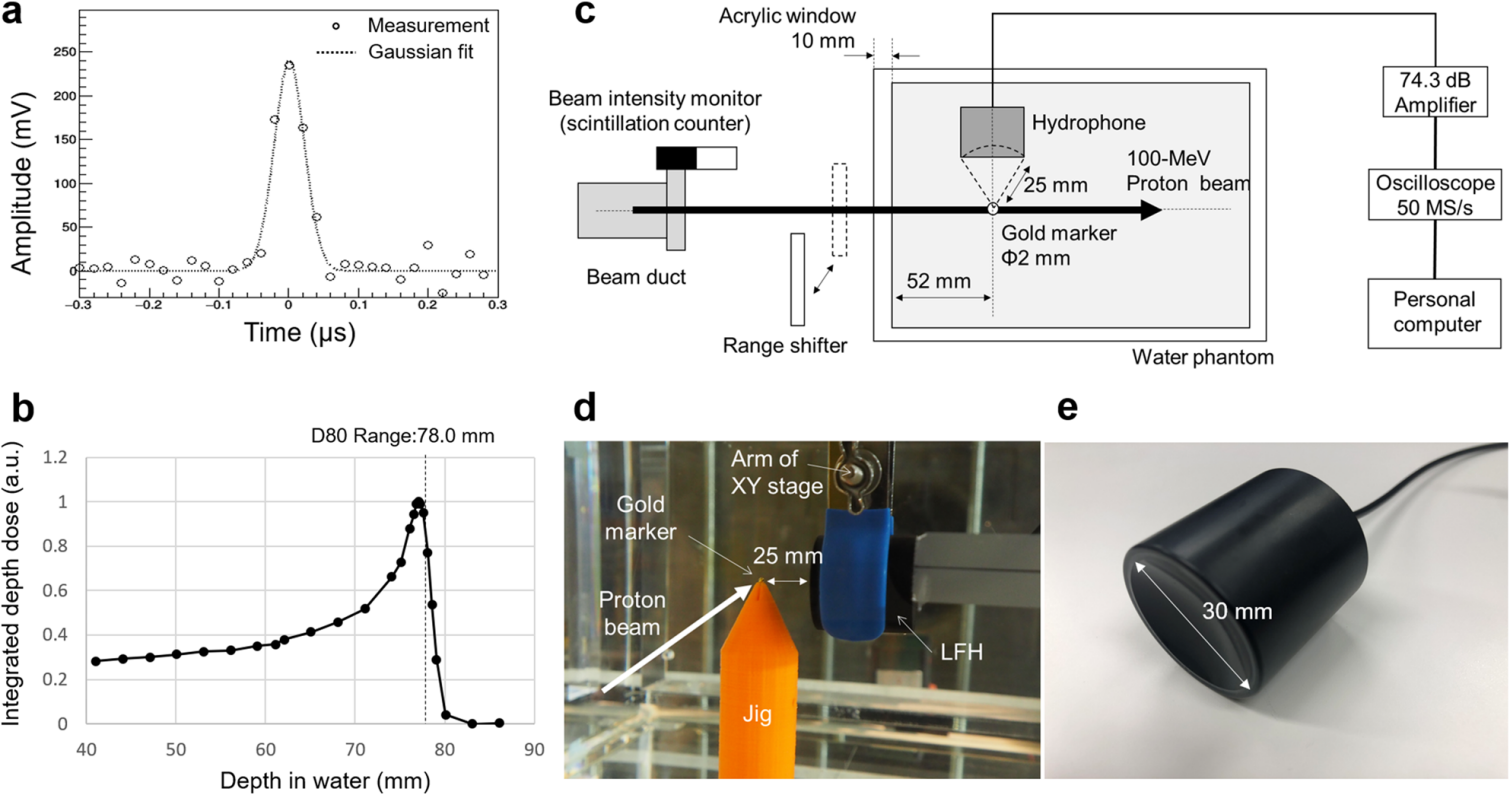
Experimental approach for observing the SPIRE. (**a**) The pulse shape as measured by a beam intensity monitor and (**b**) the Bragg curve as measured by an ionisation chamber. (**c**) Schematic of the experimental setup for the SPIRE measurement using an LFH. A 100 MeV pulsed proton beam produced by an FFA was injected into the water phantom at a frequency of 30 Hz. The temperature of the water was kept at 22 °C. A gold marker with a diameter of 2 mm was placed on the beam axis and the SPIRE emitted from the marker was collected by the LFH. The residual range at the marker was changed by stacking acrylic plates on the phantom surface. For each beam pulse, the shape of the pulse was monitored by counting the lost protons, stray neutrons and gamma-rays using an EJ-200 plastic scintillator mounted at the exit of the vacuum beam duct. For each separate run, the scintillator signal height was converted to the number of protons measured using the Faraday cup. (**d**) Photograph of the gold marker and hydrophone setup in the water phantom. A stand with a conical tip with a slit was made to support the gold marker underwater. The stand was made of ABS resin that had an acoustic impedance close to that of water. The hydrophones were mounted on an XY stage that can scan in a plane parallel to the beam direction; they were set at position where the pressure amplitude is maximized. (**e**) Photograph of the LFH. A PZT piezoelectric ceramic processed into a spherical shape was used to improve the sensitivity. The focal length was set to 25 mm to have the maximum solid angle centred at the marker while keeping a sufficient distance from the proton beam.

The experiment was conducted using the FFA at Kyoto University. A 100-MeV pulsed proton beam was generated by the accelerator using fast beam extraction^32,33^. The achieved short pulse width (1σ) of 21 ns (Fig. 2a) had a high-frequency (1.62 MHz) component of sufficiently large amplitude to generate SPIREs from the gold marker. The beam range, defined as the distal 80% dose position of the Bragg curve, was 78.0 mm according to measurements made by the ionisation chamber (described later in Sect. 4) (Fig. 2b). The beam spot size (1σ) at the surface of the water phantom was 4.9 and 5.7 mm in the vertical and horizontal directions, respectively. The beam intensity was measured with a Faraday cup, and the number of protons per pulse was found to be (1.17 ± 0.06) × 10^8^, which corresponds to ~ 19 pC and a Bragg peak dose of 0.4 Gy. The beam characteristics of the FFA were equivalent to those of scanning pencil beams used in clinical applications, except that the pulse width was several orders of magnitude smaller than that used in clinical machines and the number of protons per pulse is several times higher than the average of what is currently clinically available (eg. synchrocyclotrons reach up to ~ 5 pC / pulse^34^).

As shown in Fig. 2c, d, a spherical gold marker with a diameter of 2 mm was placed in a water phantom lying on the beam axis. Data were collected using two different hydrophones independently. A large spherically focused hydrophone (LFH) comprising a piezoelectric ceramic was used to increase the sensitivity (Fig. 2e). This LFH had been specially customised to have a central frequency of 1.62 MHz and a focal distance of 25 mm. No matching layer was used to increase the sensitivity at the resonance frequency. The LFH was placed at the focal distance from the marker, lateral to the beam axis. SPIREs were measured with varying *R*_*res*_ at the marker—this was achieved by stacking acrylic plates in front of the water phantom. Although the LFH had a high sensitivity, no calibration protocol to obtain the absolute pressure from the observed raw signal (voltage) has yet been established. The second hydrophone was, thus, used for this reason. The second hydrophone was a V397-SU (Olympus Corporation, Tokyo, Japan), which is commercially available and has a central frequency of 2.25 MHz. The signal amplitude obtained by this hydrophone is smaller than the LFH since it is an unfocused type and uses a matching layer. However, since the pressure-voltage conversion coefficient can be estimated by the cross-calibration procedure (see Sect. 4), it could be used to roughly evaluate the absolute value of the pressure field.

### Experimental results

#### SPIRE generation from a spherical gold marker

**Figure 3:**
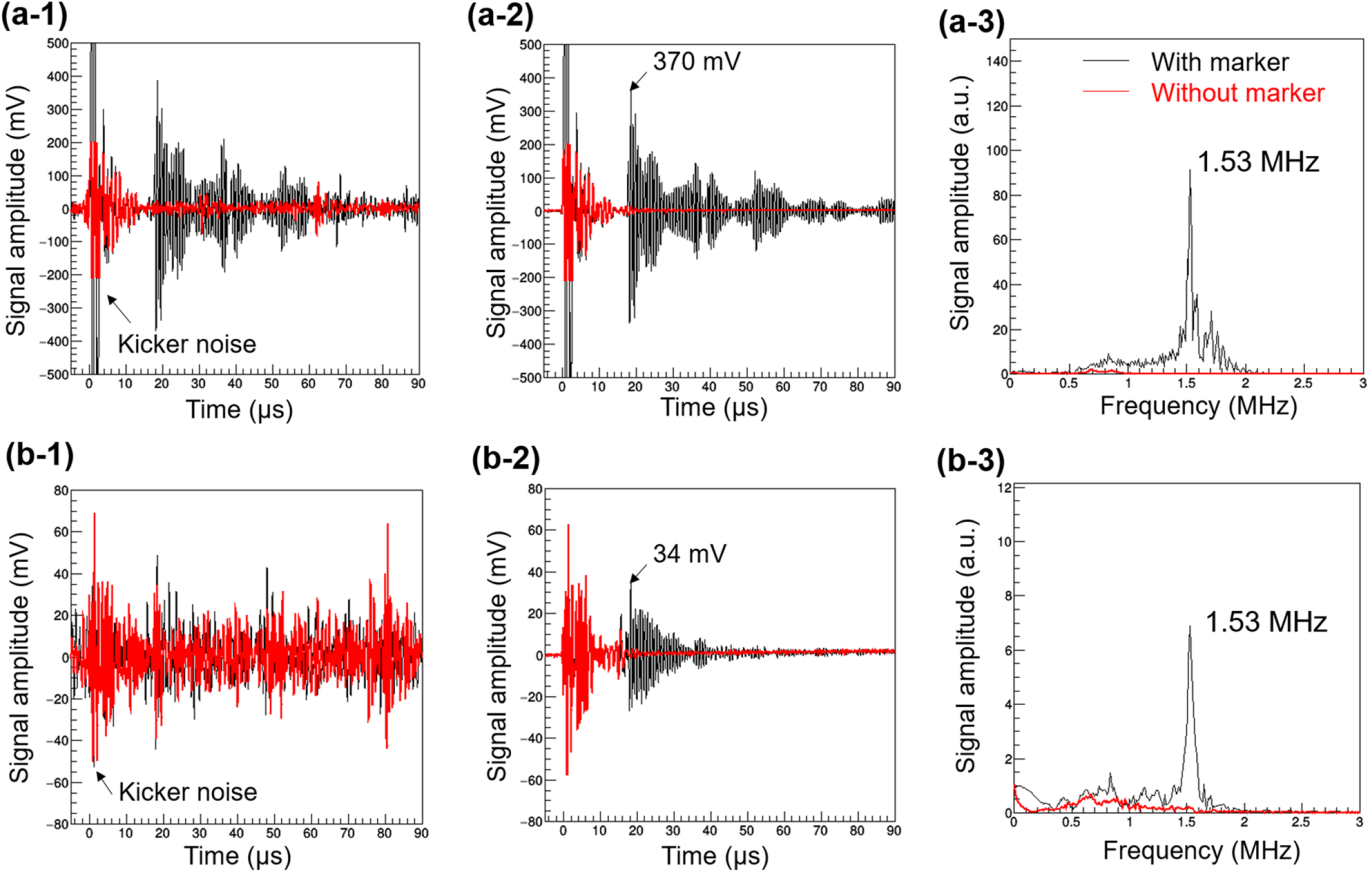
Measuring the waveforms and frequency spectra of a SPIRE. SPIRE waveforms obtained by (**a**) the LFH and (**b**) the V397-SU. Data from a single measurement (left-hand column) and the results of averaging over 1000 measurements (central column) are shown. Time zero represents the time of irradiation by the beam. The black and red lines denote the results obtained with and without the spherical gold marker, respectively. In the time domain, both detectors detected a characteristic resonance signal about 17 μs after irradiation, which did not exist when the gold marker was not present. The strong signal observed around time zero is the electromagnetic noise emitted from the beam extraction kicker of the FFA: this was observed regardless of the presence or absence of the gold marker. The frequency spectra (right-hand column) were obtained from the Fourier transform of the averaged waveforms shown in the central column. To eliminate the influence of the kicker noise, waveforms from 15 to 90 μs after beam irradiation were analysed. Although the waveforms shown in (**a**) and (**b**) appear different in the time-domain due to the structural differences between the detectors, pronounced peaks were observed at 1.53 MHz in the spectra using both detectors. This is close to the theoretical prediction of 1.62 MHz.

Figure 3 shows the observed SPIREs at a fixed *R*_*res*_ of about 14 mm. Figure 3a-1 and a-2, respectively, show the waveform for a single measurement and the average waveform from over 1000 measurements, as acquired by the LFH. Figure 3b-1 and b-2 show the waveforms that were acquired by the V397-SU. Figure 3a-3 and b-3 show the frequency domain data corresponding to the waveforms in (a-2) and (b-2), respectively. As shown in Fig. 3a-1, a-2, and b-2, characteristic resonance signals, which were not observed when the gold marker was not present (the red line shows the results obtained without the marker), appeared at approximately 17 μs after irradiation of the beam in both detectors. This arrival time agrees well with the value (16.8 μs) calculated based on the velocity of sound in water (1489 m/s at 22 °C) and the distance between the centre of the marker and hydrophone (25 mm). As shown in Fig. 3a-3 and b-3, in both detectors, the peak frequency of the waveforms was 1.53 MHz, which is close to the theoretically predicted frequency (1.62 MHz) but 0.1 MHz smaller. This difference can probably be attributed to the slight deformation of the marker. As described in Sec. 1, the lowest frequency of the SPIRE is $f=c/\phi$ where $\phi$ is the marker diameter (2 mm) and *c* is the sound velocity. $f$ of 1.53 MHz is realized when $\phi$ is 2.1 mm (0.1 mm greater than the nominal size).

Remarkably, the signal measured by the LFH was sufficiently large (~ 370 mV at maximum) compared to the background noise (~ 30 mV), and thus, averaging was not required to identify the SPIRE (Fig. 3a-1 and a-2). This is an attractive feature for clinical applications. In contrast to the SPIRE waveform observed by the V397-SU, which decayed quickly and monotonically, the waveform observed by the LFH was found to decay slowly in the form of a modulated envelope with nodes. This modulated envelope may have resulted from the occurrence of vibration modes other than the longitudinal mode in the piezoelectric ceramic or may have been caused by imperfect shielding. In addition, due to the lack of a matching layer, part of the SPIRE was reflected between the piezoelectric ceramic surface and the gold marker, producing repeated waveforms 34 μs after the first arrival of the SPIRE. Nevertheless, as shown below, the correlation between the amplitude of the SPIRE and the residual range took the same form as that predicted by the simulation.

#### Correlation between SPIRE amplitude and residual range

**Figure 4:**
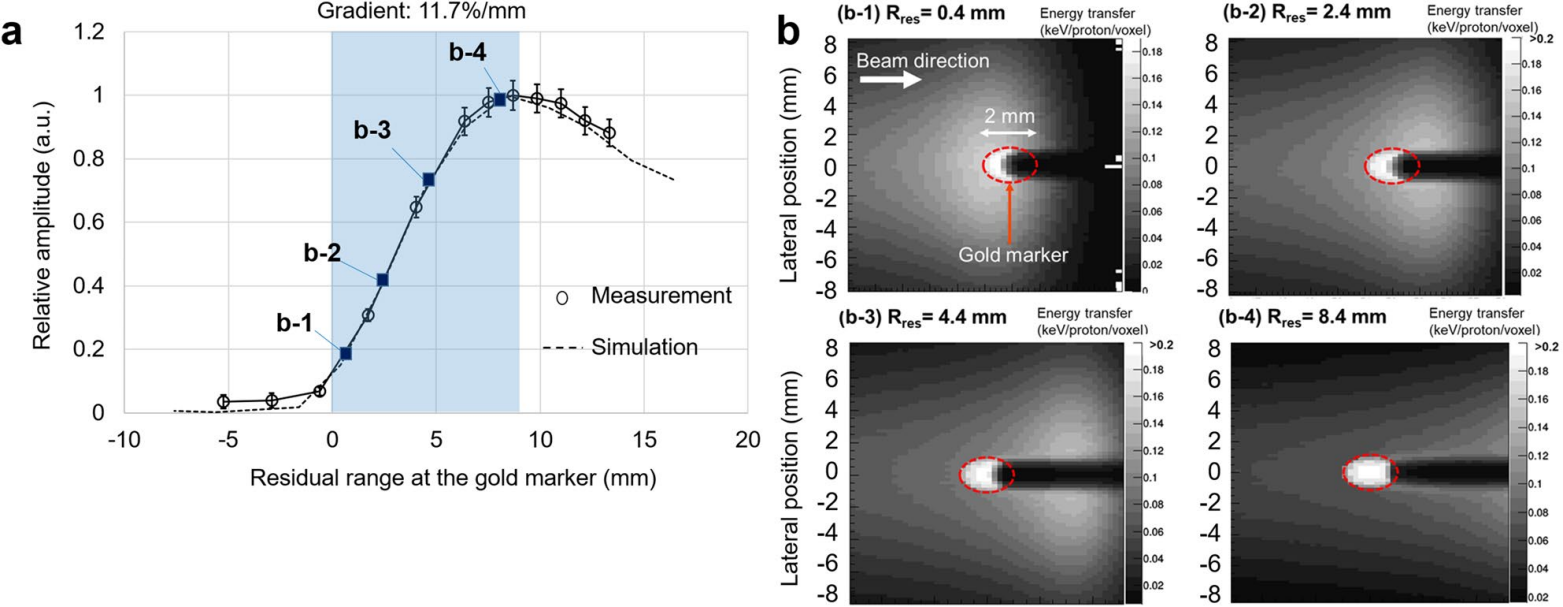
Variation in SPIRE amplitude with the residual range at the marker position. (a) The SPIRE amplitude, defined as the amplitude at the peak frequency (1.53 MHz), as measured for different values of the residual range at the marker by stacking the acrylic plates in front of the water phantom. (b) For each residual range (*R*_*res*_ of 0.4, 2.4, 4.4, and 8.4 mm), the calculated energy distributions in and nearby the gold marker are shown in (b-1)–(b-4), respectively. As shown in (a), the measurement results agree well with the results of the simulation after the application of a systematic correction of 0.8 mm to the measured residual range. The peak frequency for the simulation was 1.62 MHz. A sharp increase in the SPIRE amplitude (11.7%/mm) was observed for 0 mm < *R*_*res*_ < 9 mm (the region shaded in blue), which reflects the strong variation in the size of the energy deposition in the marker around the Bragg peak (shown in (b)). Given the relatively small SD of the signal, the beam range can be estimated with submillimetre accuracy as long as this experimental setup is used.

Figure 4a shows the SPIRE amplitude at resonance in the frequency domain (1.53 MHz), as measured by the LFH and plotted in terms of *R*_*res*_. Here, the value of *R*_*res*_ for each data point was derived from the marker position and the beam range measured by the ionisation chamber (see Sect. 4 for more details), and then corrected for a systematic error of 0.8 mm. This error could have occurred during the positioning process of the gold marker and the ionisation chamber. Since they are positioned with the guidance of a laser marker whose line width is about 3 mm, the poisoning error can be as large as 0.5 mm each and this leads to the total systematic error of 0.8 mm in the experiment. The amplitude plotted on the vertical axis has been normalised using the maximum amplitude achieved at *R*_*res*_ of 9 mm, and the error bars represent the standard deviation (SD) over 1000 measurements.

As shown in the plot, the measured data agree well with the curve obtained from simulations, with the simulation lying within the error bars. For *R*_*res*_ < 0 mm, the SPIRE amplitude goes to almost zero, other than for the noise, since no dose was received by the marker in such cases. At 0 mm < *R*_*res*_ < 9 mm (the blue shaded region in Fig. 4a), the SPIRE amplitude increases quite sharply (at 11.7%/mm) and is approximately linearly correlated with *R*_*res*_. This reflects the strong variation in the amount of energy deposited in the marker close to the Bragg peak (Fig. 4b-1–b-4). As the reproducibility of the SPIRE measurements is relatively high and the SD is only 4.3% at most in this region, the beam range can be estimated with an accuracy of less than 0.4 mm as long as this experimental setup is used. When *R*_*res*_ > 9 mm, the SPIRE amplitude is negatively correlated with *R*_*res*_. Since the slope is less steep, the range could not be predicted with submillimetre accuracy in this regime.

### Simulation study

**Figure 5:**
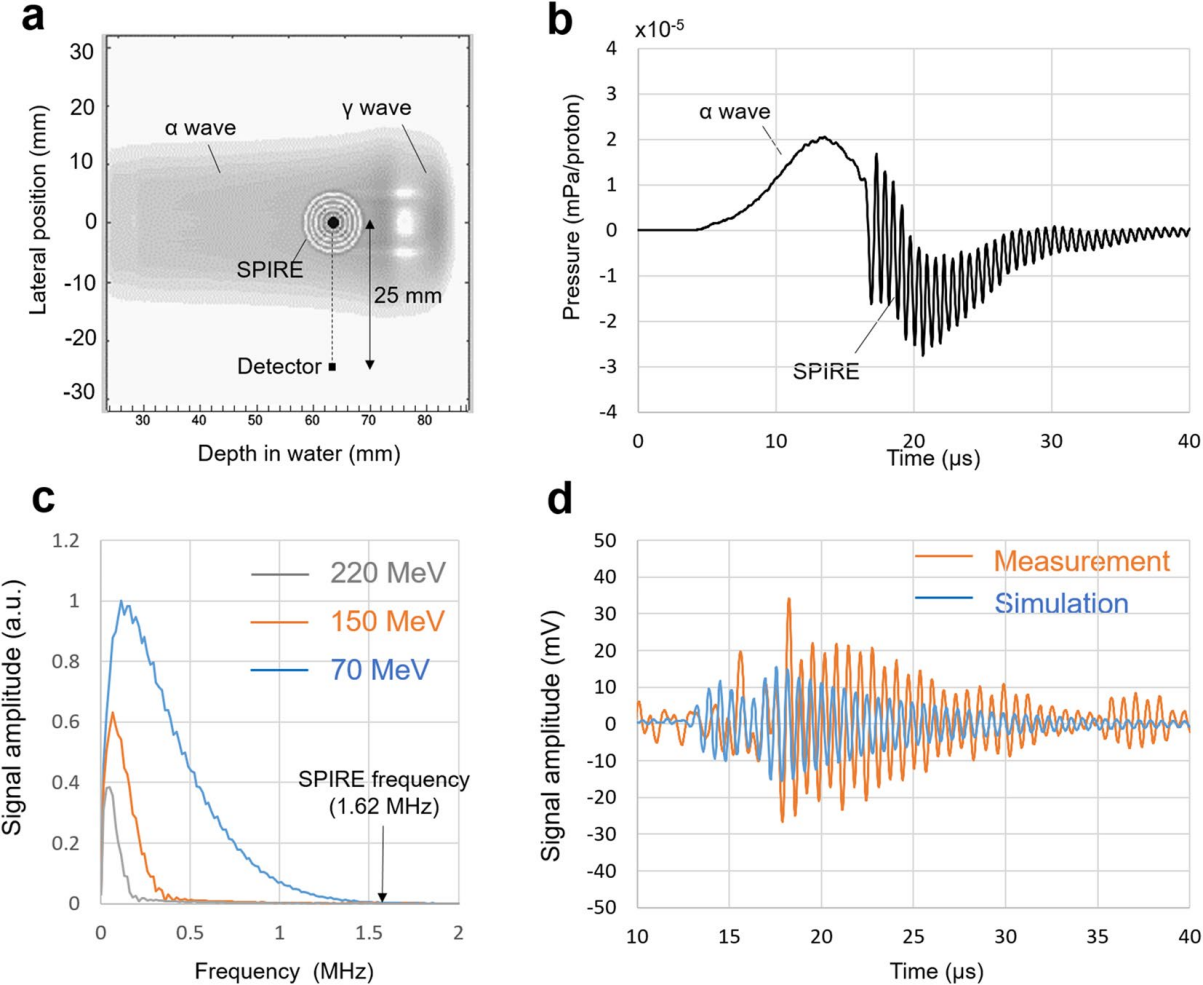
Simulations of the acoustic waves and comparisons with the measured waves. (**a**) Ionoacoustic wave propagation simulation showing three characteristic waves that appear after the beam injection: a wave from the plateau region of the Bragg curve (α-wave), a wave from the Bragg peak (γ-wave) and a SPIRE emitted from the marker. Higher pressure areas are shown in darker colours. The beam energy was 100 MeV (the beam range of 78.0 mm) and the beam spot size (1σ) at the surface of the water phantom was 4.9 and 5.7 mm in the vertical and horizontal directions, respectively. The beam pulse width (1*σ*) was set to 100 ns in this simulation. (**b**) The pressure waveform detected by a point virtual detector placed at 25 mm from the marker. The detector response was assumed not to depend on the frequency. The pressure amplitude of the α-wave was of the same order as that of the SPIRE. (**c**) The frequency spectra of the γ-waves detected by a point virtual detector placed on the beam axis at 20 mm downstream of the beam range. The simulation was performed with three proton energies (70, 150 and 220 MeV) used in clinical machines. The maximum frequency of the γ-waves was less than 1.5 MHz and thus the separation of the SPIRE from the γ-wave is always possible. (**d**) The measured and simulated SPIRE: the results are similar but an absolute difference in the pressure amplitude was observed. The simulated waveform was obtained after including the detector size effect and applying the amplified and bandpass-filtered frequency response of the V397-SU hydrophone.

Figure 5 shows the results of the acoustic wave simulation (see Sect. 4 for more details) conducted using the pulse temporal structure and dose distribution. As shown in Fig. 5a, originally, three types of ionoacoustic waves were generated from the energy deposition: an α-wave from the plateau region of the Bragg curve, a γ-wave from the Bragg peak^35,36^ and the SPIRE. The detector in our experiment was set lateral to the beam axis, where the α-wave and the SPIRE arrived together at the position of the detector (Fig. 5b). The frequency of the α-wave was less than 150 kHz, which allowed us to separate the SPIRE from the α-wave by applying a bandpass filter. The key factor in determining the frequency distribution of the α-wave was the beam spot size, which was about 5 mm (1σ) in this setup. In clinical machines, the spot size typically ranges from 2 mm to less than 10 mm depending on the beam energy and depth. Therefore, the frequency of the α-wave never exceeds 1 MHz for all beam energies used in clinical settings (typical values are 70–220 MeV). Thus, the extraction of the SPIRE will always be possible.

In contrast, when the detector was placed downstream of the Bragg peak as in a previous study^10^, the γ-wave more strongly affected the SPIRE measurement than the α-wave. The simulation was extrapolated to cover the energy range used in clinical proton therapy (70–220 MeV), and this revealed that the maximum frequency of the γ-waves was less than 1.5 MHz, implying that, again, extraction of the SPIRE is possible using bandpass filtering (Fig. 5c). Compared to the α-wave, the γ-wave contains a frequency component closer to the resonance frequency. Therefore, placing the detector perpendicular to the beam axis during the SPIRE measurement is more appropriate. The extraction of the SPIRE from other waves (α- and γ-waves) is expected to be possible for other pulse width and pulsing structures. This is because the pulse structure exploited in this study is almost impulse and reducing the pulse rising time or pulse width does not change the frequency spectra of the α- and γ-waves. On the other hand, increasing the rise time or pulse width leads to the decrease in frequency of these waves, causing the increase of the frequency gap from the SPIRE.

As shown in Fig. 5d, by applying the frequency response function of the V397-SU hydrophone and including the detector size effect, a simulated waveform similar to that observed in this experiment was obtained. As with the case of LFH, the slight difference of the waveform might be due to the occurrence of vibration modes other than the longitudinal mode in the piezoelectric ceramic or may have been caused by imperfect shielding. In the future, reduction of the difference between these waveforms may be possible by including detailed information about the structure and composition material of the hydrophone in the simulation; however, this is not possible at present. In addition, as denoted in Sec. 4, we assumed a parallel beam flux at the surface of the water phantom in the simulation. This simplification may have caused the deviation in the magnitude of wave amplitudes between the simulation and experiment. Increasing the accuracy of the conversion from the signal voltage to the pressure will also help reduce this difference^21^. In clinical PBT applications, further improvements in the accuracy of the simulation will be required to allow a prediction of the correlation between the SPIRE amplitude and residual range to be made in advance of the beam delivery.

## Discussion

The experiment depicts a proof-of-principle demonstration of the proton beam range detection method based on the SPIRE emitted from a biocompatible gold marker that was the same as those clinically used for patient positioning. As mentioned in a previous study^24^, the gold marker can be regarded as a new type of *in-vivo* point dosimeter. Compared to other kinds of *in-vivo* dosimeters, such as implanted wireless dosimeters^37^, and PET imaging of radioactive markers^38^, this technique achieved an equivalent range-detection performance (≤ 1 mm), but it is more advantageous since it does not require any additional devices to be inserted into the patient’s body. A common limitation for these implanted dosimeters is that the beam range is determined only for a limited number of spot beams, i.e., “key spots”, that pass through the dosimeters. To make maximum use of these spots and increase the SPIRE amplitude, the intensity modulated proton therapy (IMPT) technique^39^ may be suitable in which a high dose is delivered to the key spots that are used for the SPIRE measurement. Note that this idea of “key spot” was already proposed in Tian et al. by the name of “SubPencilBeam” in the context of prompt gamma imaging^40^. The dose homogeneity is then recovered by suppressing the beam intensity delivered to the surrounding spots.

We observed a sizeable pressure wave using a customised hydrophone and FFA which can generate very short pulsed and high intensity beam; however, there is a significant scope for the further development of this technique for use in clinical applications. The relatively high resonance frequency of 1.62 MHz (equivalent to a wavelength of 1 mm in water) indicates that the signal amplitude varies by a significant amount for a small shift in the hydrophone position. For the LFH, a shift of only 0.2 mm causes a 10% decrease in signal amplitude, which is unfavourable for clinical applications. Reduction of the detector size should improve the robustness of the technique to the positioning error but at the expense of a large signal-to-noise ratio. State-of-the-art optical ultrasound detection could be a promising candidate for replacing the piezoelectric hydrophone: it has a smaller sensing-element size of less than 1 mm^2^, and its signal-to-noise ratio is much greater than that of piezoelectric hydrophones^41^. We also note that the beam-positioning error affects the signal amplitude. However, the lateral position of the beam is continuously monitored and is measured to an accuracy of within 0.5 mm using current commercial clinical machines^42^. This means that the error in the range estimation is 0.8 mm if the lateral beam spot size is 5 mm (1σ) and the gold marker is located 1σ away from the centre of the beam spot. Improving the accuracy of the wave simulation is also necessary for clinical applications. As mentioned above, including detailed structural and material information about the hydrophone in the simulation as well as accurately deriving the piezoelectric constant is essential. In addition, for clinical applications, the tissue acoustic properties (eg. sound velocity and frequency-dependent acoustic attenuation) and tissue heterogeneity have to be considered accurately in the wave propagation modelling. Since the current knowledge of the acoustic properties for biological tissue is limited, the estimated range has to be considered with caution. The effect of tissue heterogeneity may be incorporated by using the CT image in which the tissue property is assigned to each voxel^14^. However, the quantitative assessment based on heterogeneous phantoms and small-animal experiments are essential and required before this range verification method is applied in clinics.

This proof-of-principle experiment was designed to demonstrate the feasibility of using SPIREs for range detection in spot-scanning proton therapy. Not only the customised hydrophone, but also the FFA performance contributed the SPIRE emission and the signal enhancement. The main difference between the beam produced by an FFA and those used in current clinical machines is the pulse width^13^. While the two types of accelerators (cyclotron and synchrotron) currently used in clinics provide a direct-current beam with a pulse length of several milliseconds, an FFA has a pulse length of about 20 ns, which is necessary for producing a SPIRE with a reasonably large amplitude. Many studies have investigated the use of an FFA in PBT mainly because it can achieve high beam intensities and also because it can switch off the accelerating cavity when the required energy has been obtained, thus allowing extraction at arbitrary energies^31^. The results described in this study may imply that an additional advantage, namely the capability for ‘on-line range verification’, can be added the list of advantages of an FFA. For the clinical implementation of FFA, however, further studies are required to suppress the fluctuations of circulating charge in accelerators as well as the variation of beam extraction efficiency among pulses so that one can control the delivered dose accurately even with the high beam intensity.

In summary, a technique for realising proton-beam range detection with submillimetre accuracy has been described, which exploits the SPIRE emission from a spherical fiducial marker. In a proof-of-principle experiment using short-pulsed proton beams at a clinical dose of 0.4 Gy, we demonstrated that the Pascal order pressure wave generated from the marker and the correlation between the beam range and the SPIRE amplitude agreed well with the predictions of numerical simulations. The detection system was focused on the resonance frequency, and this allowed us to estimate the beam range from a single measurement. The development of a detection system that is robust to misalignment is necessary for clinical applications, and this will be the subject of our future work.

## Methods

### Data acquisition system

The SPIRE emitted from the marker was collected by two hydrophones (LFH and V397-SU). The signal was amplified by the 74.3 dB charge sensitive preamplifier with bandpass filter centred at the resonant frequency (1.62 MHz). HDO6104A (Teledyne LeCroy., New york, US) with 12 bit analog-to-digital converter was used for the data logger. The signal was stored at a 50 MS/s sampling rate. A control signal for the beam extraction kicker of the FFA was used as the data acquisition trigger.

### Pressure–voltage conversion for the V397-SU

To derive the pressure–voltage conversion coefficient for the V397-SU hydrophone, the detector was cross-calibrated against a needle-type hydrophone—an HPM05-S (Precision Acoustics Ltd., Dorchester, UK)—for which the sensitivity values are provided by the National Physical Laboratory in UK. For a plane acoustic field, the conversion coefficient was estimated to be 7.35 V/MPa at 1 MHz. Based on the frequency response and after considering the difference between the types of acoustic fields (plane versus spherical), we estimated the conversion coefficient for the 1.62-MHz spherical waves to be 2.1 V/MPa.

### Residual range measurement

We obtained the residual range at the gold marker’s position in Fig. 4 a from measurements of the Bragg curve (Fig. 2b) made using a type-34070 Bragg peak chamber (PTW–Freiburg, Freiburg, Germany). This is a plane-parallel chamber that is commonly used in PBT dosimetry. The distal 80% position of the Bragg peak (a depth of 78.0 mm) was identified as the beam range. The residual range was derived by subtracting the thickness of the acrylic wall (10 mm) and plates (1–16 mm) and also the distance between the gold marker and the inner surface of the water phantom (52 mm).

### Simulation of the transferred energy distribution

The spatial distribution of the energy transferred by the proton beam was simulated by Geant4 (version 9.3)^43^ and was used as the input of the acoustic wave transport simulation described below. Geant4 simulates the transport of protons and their interactions with matter using the Monte Carlo method. The input beam parameters, including the mean energy of the incident protons, energy spread and the beam size at the surface of the water phantom, were tuned to reproduce the shape of the Bragg curve that was obtained using the above-described Bragg peak chamber and to achieve the lateral beam profiles that were obtained by a radiochromic film (GAFchromic EBT3: Ashland Inc., Covington, USA) attached to the surface of the water phantom. A Satera MF8570Cdw (Canon Inc., Tokyo, Japan) was used as the film scanner. For simplicity we assumed the momenta of protons were parallel to the beam axis at the surface of the water phantom whereby the beam size is the only parameter to be validated in the phase space. For the physical models, the configuration from the Standard Physics List option 3 was used for the electromagnetic processes, and the G4BinaryCascade and G4HadronElastic models were used for the inelastic and elastic hadron processes, respectively. The cut-off range used for the secondary electrons, positrons and photons was 1 mm. The grid size used for the calculations was set to 0.2 mm × 0.2 mm × 0.2 mm to accurately represent the shape of a spherical marker with a diameter of 2 mm. A total of 10^7^ proton histories were tracked to achieve sufficient statistics.

### Simulation of SPIRE emission and propagation

The simulation of the SPIRE emission and propagation was conducted using k-Wave toolkit, which is a MATLAB toolbox that can be used to calculate the propagation of acoustic wave in the time domain^44^. This code basically solves the coupled first-order differential equations for the acoustic particle velocity and acoustic density. The acoustic source term was expressed as $\left(\Gamma /{v}^{2}\right)E\left(\overrightarrow{r}\right)G\left(t\right)$, where $E\left(\overrightarrow{r}\right)$ is the transferred energy distribution calculated using Geant4, $G\left(t\right)$ is the pulse structure and $\Gamma$ and $v$ are the Gruneisen coefficient and the velocity of sound in the medium, respectively. A perfectly matched layer was applied to the boundary to absorb all outgoing acoustic waves and prevent reflection. The dimensions of the calculation grid were again set to 0.2 mm × 0.2 mm × 0.2 mm to accurately represent the shape of a spherical marker with a diameter of 2 mm as well as to accurately simulate the propagation of high-frequency waves. The time step was set to 30 ns to ensure stability (Courant–Friedrichs–Lewy number < 1) while suppressing the calculation memory. As shown in Fig. 2a, the measured beam pulse width (1σ) was 21 ns; this was set to 100 ns in the numerical calculation because the calculation time step was 30 ns and too coarse to represent the measured pulse. According to our previous study^21^, the calculated amplitude of the SPIRE for a beam pulse width of 21 ns is 1.64 times larger than that for a beam pulse width of 100 ns. Thus, a correction factor of 1.64 was used to calculate the pressure values shown in Fig. 5d.

## Data Availability

Data that support the findings of this study are available from the corresponding author upon reasonable request.
